# Biocomposite Inks for 3D Printing

**DOI:** 10.3390/bioengineering8080102

**Published:** 2021-07-22

**Authors:** Gary Chinga-Carrasco

**Affiliations:** RISE PFI, Høgskoleringen 6b, 7034 Trondheim, Norway; gary.chinga.carrasco@rise-pfi.no

Three-dimensional (3D) printing has evolved massively during the last years and is demonstrating its potential in tissue engineering, wound dressings, cell culture models for drug testing, and prosthesis, to name a few. One important factor is the optimized composition of inks that can facilitate the deposition of cells, fabrication of vascularized tissue and the structuring of complex constructs that are similar to the human micro-environment or functional organs. Some of the key aspects regarding the formulation of bioinks (inks biocompatible with cells) are, e.g., the tailoring of mechanical properties of the supporting matrix, biocompatibility considering the targeted tissue and the rheological behavior of the ink which may affect the cell viability, proliferation and cell differentiation. Biocomposite inks can include several polymers, such as polyhydroxyalkanoates, polylactic acid, collagen, agarose, alginate, nanocellulose, and may be complemented with cross-linkers to stabilize the constructs and with bioactive molecules to add functionality. Hence, these topics were covered by this Special Issue, which was supported by international groups with key competence in these areas of research and development.

The advances regarding the regeneration of functional tubular tissues and organs were explored by Jeong et al. [1]. The authors described several technologies such as extrusion-based, inkjet, laser-assisted and stereolithography-based bioprinting, and considering relevant inks, based on collagen, gelatin, alginate and synthetic polymers. The limitations of traditional methods to fabricate shape-free structures were mentioned, emphasizing the applicability of free-shape constructs which are based on indirect 3D printing, i.e., a hydride system where a 3D mold is printed to indirectly form 3D constructs. According to the authors, extrusion-based systems (also called direct-ink writing) are flexible and appropriate for fabrication of tubular structures. However, tubular structures such as the esophagus, blood vessels, and trachea are still demanding to fabricate and apply as clinical substitutes due to various physiological aspects [1].

Fused deposition modelling (FDM) is an extrusion-based technology, where a melted polymer is deposited layer by layer in predefined x,y,z locations. There are various polymers that can be applied for FDM 3D printing and the most applied is polylactic acid (PLA). However, natural polymers such as polyhydroxyalkanoate (PHA) are becoming an interesting but still limited alternative within the area of 3D printing and in the biomedical field [2]. PHA is naturally produced by microorganisms such as bacteria and archaea. Due to the versatility of the polymer various application areas were mentioned by Giubilini et al., including drug delivery, vessel stenting, tissue engineering, emphasizing also the opportunities offered by 3D printing such as the fabrication of non-toxic, resorbable scaffolds for tissue regeneration [2].

Collagen, which is found in the extracellular matrix (ECM), is another natural polymer that has been utilized for decades to enhance cell cultures, and more recently as a biomaterial for 3D bioprinting and tissue engineering [3]. The popularity of collagen is exemplified by the great number of commercial products currently available. However, the poor mechanical properties of collagen are mentioned as a limitation of the biomaterial, and the authors provide some strategies for chemical and physical cross-linking to counteract this limitation [3]. The authors also dedicated a section to regulatory considerations which is interesting and valid also for other biomaterials and products. Furthermore, the hydrolysis of collagen leads to the formation of gelatin that can be combined with alginate to form a biocomposite ink for the 3D printing of scaffolding material. This was successfully demonstrated by Somasekharan et al. [4], where a hydrogel composed of alginate dialdehyde, gelatin and platelet-rich-plasma was formulated to 3D bioprint cross-linked cell-laden constructs with 80% cell viability. Agarose is another linear polysaccharide that has been reported to form constructs with high stiffness [5]. The agarose was modified (by TEMPO mediated oxidation) and combined with minor amounts of native agarose to form a biocomposite ink for 3D printing by micro-extrusion. The authors demonstrated the potential of the biocomposite ink by printing a series of complex and impressive 3D self-standing shapes.

The application of bacteria in the area of 3D printing and biofabrication was reviewed by Shavandi and Jalalvandi [6]. The authors mentioned the printing of bacteria aided by polymers such as gelatin and alginate and cross-linked with calcium to stabilize the constructs. Such systems may find their application area in the biofabrication of model biofilms containing a predefined distribution of bacteria such as *Pseudomonas aeruginosa* and *Staphylococcus aureus* which may be used to test antibacterial agents for, e.g., treatment of infected wounds where these bacteria are common pathogens.

Cellulose fibers have been explored for years as reinforcement of polymers to form biocomposites. Cellulose fibers can also be mechanically processed to obtain microfibrillated cellulose. In this Special Issue, a timely application of a polypropylene/microfibrillated cellulose biocomposite was demonstrated in the 3D printing of prosthetic products. The main advantage of this approach is the possibility to 3D print medical devices that are tailor-made for individual patients. This was successfully demonstrated by Stenvall et al. [7], where a transtibial prosthesis was 3D printed by FDM. The prosthesis was tested by patients and clinicians, and positive feedbacks were obtained regarding the use of a biocomposite product instead of conventional prosthesis [7]. Cellulose can also be processed chemically to obtain a printable gel [8]. The cellulose gel (20 wt%) was tested extensively following statistical approaches to find the best parameters for optimal 3D printing. The authors demonstrated the printability of the gel by firstly printing simple cubes and then more complex structures such as ear models [8].

Nanocellulose is one of the most recent biomaterials that have entered the 3D printing space. The shear-thinning property of nanocellulose is most appropriate for 3D printing by micro-extrusion systems. The stiffness of pre-defined 3D constructs can be tailored for the targeted tissue [9]. Wang et al. [9] reviewed several aspects of nanocelluloses, including the impact of surface charge and modification on, e.g., cell survival, cell attachment and proliferation. However, according to the authors, aspects that still require attention are the control of biodegradability in the human body and potential nanotoxicity, which are also considered major topics of research by the scientific community.

There are several types of nanocelluloses that can be obtained by various pre-treatments [9]. Enzymes are also applied in the pre-treatment step in order to facilitate the nanofibrillation. Kangas et al. [10] demonstrated the production of unbleached and delignified nanocelluloses based on an enzymatic pre-treatment, and their potential suitability as ink for 3D printing. The study demonstrated that the enzymatic pre-treatment was more effective on the delignified pulp and that an additional fluidization step was required to secure a nanocellulose grade with adequate morphology and rheology for 3D printing by micro-extrusion systems. The authors proved that the material was not cytotoxic and could be used to print self-standing 3D constructs.

Espinosa et al. [11] demonstrated the application of biocomposite inks (containing TEMPO nanocellulose, varying amounts of alginate and cross-linked with Ca^2+^), for wound dressings. Wound care causes a significant economic burden on patients and healthcare systems; thus, research on advanced wound dressings that could be tailor-made by 3D printing has been a major area of research during the last years. In this specific study, TEMPO nanocellulose-based inks performed well in 3D printing operations and the 3D printed constructs had in addition great capacity to maintain water [11], which are considered beneficial characteristics for novel and personalized wound-dressing devices.

Research on cancer, one of the most abundant diseases worldwide, would benefit from developing relevant tissue-mimicking micro-environments in order to test new drug candidates. Rosendahl et al. [12] reported on an extensive study, including gene expression analysis, and showed the effect of 3D printed TEMPO nanocellulose scaffolds on cancer cells, as a step to develop novel tumor model systems. The analysis demonstrated that 3D printed nanocellulose scaffolds induced cancer stem cell characteristics on both genetic and cellular levels [12]. In addition, a heterogenous cell population was revealed, growing in multiple layers mimicking the in vivo situation in contrast to conventional 2D cell cultures where cells grow in a monolayer with a homogeneous cell population. The authors concluded that carboxylated nanocellulose represents a promising material for 3D cell culture models for cancer applications and drug screening.

In summary, the studies included in this Special Issue cover a vast area of research and provide clear examples of 3D printing technologies and applications, also emphasizing the benefit of additional converging technologies, such as chemical engineering, nanotechnology, biotechnology and gene sequencing. It is expected that these technologies, combined with artificial intelligence and advances in gene editing will lead to exponential growth and further disruption of this fascinating area of research, with a main focus on the manufacturing of physiologically relevant and functional bioconstructs.
